# An Innovative Serum With Retinol, Hydroxypinacolone Retinoate, Peptides, and Silybin Improves Mild Photoaged Facial Skin in Middle‐Aged Chinese Women

**DOI:** 10.1111/jocd.70627

**Published:** 2025-12-25

**Authors:** Yixin Shen, Muyang Shi, Ying Ye, Chenlan Xu, Xiao Feng, Tianyu Bi, Yuyan Chen, Jiefang Huang, Yanan Li, Peiwen Sun

**Affiliations:** ^1^ Research & Innovation Center Proya Cosmetics Co. Ltd. Hangzhou China

**Keywords:** anti‐aging, Asian skin, HPR, peptides, photoaging, retinol, silybin, skincare formulation, TGF‐β/Smad signaling

## Abstract

**Background:**

Retinoids are effective anti‐aging agents, but their use is often limited by irritation, particularly in Asian skin. Hydroxypinacolone retinoate (HPR), a novel retinoid ester, offers similar benefits with reduced irritation, yet its combined use with retinol remains poorly characterized. Additionally, the integration of peptides and botanical antioxidants like silybin into retinoid‐based regimens lacks mechanistic and clinical evidence.

**Aims:**

To investigate the molecular mechanisms and clinical efficacy of a novel serum containing retinol, HPR, peptides, and silybin in improving mild photoaged facial skin in Chinese women.

**Methods:**

Mechanistic effects were investigated using transcriptomic profiling in a keratinocyte–fibroblast co‐culture and protein expression analysis in UV‐exposed ex vivo skin models. Clinical efficacy was assessed in an 8‐week study in middle‐aged Chinese women with mild photoaging.

**Results:**

Retinol + HPR synergistically activated TGF‐β/Smad signaling and enhanced extracellular matrix gene expression compared with retinol alone. The addition of silybin further promoted collagen and elastin synthesis. Clinically, the serum significantly improved wrinkles, elasticity, hydration, barrier function, and pigmentation with excellent tolerability.

**Conclusions:**

This multi‐active serum demonstrates a synergistic, well‐tolerated anti‐aging effect. To our knowledge, this work provides an integrated molecular‐to‐clinical evaluation of a retinol‐HPR‐peptide‐silybin combination, supporting its potential as a potent yet gentle anti‐aging option for sensitive skin.

## 1 Introduction

Photoaging, the premature aging of skin caused by chronic ultraviolet exposure, is characterized by wrinkles, loss of elasticity, uneven pigmentation, and rough texture [1]. Topical retinoids (vitamin A derivatives) are widely recognized as effective treatments for photoaging, promoting epidermal turnover and dermal collagen production [2]. Retinol, the cosmetic form of vitamin A, remains a gold‐standard ingredient for improving fine lines and skin texture. However, retinoid treatments frequently induce irritation—dryness, erythema, peeling—which limits their broader use, especially in Asian populations [3, 4]. Asians tend to be more sensitive to retinoids than Caucasians, leading to recommendations for lower concentrations or less frequent application. Indeed, a recent study in Chinese women found that 0.1% retinol (in a specially formulated serum) improved mild photoaged skin with good tolerability [5, 6]. There is thus a strong need to optimize retinoid‐based therapies to retain anti‐aging efficacy while reducing irritation.

Hydroxypinacolone retinoate (HPR) is a newer‐generation ester of all‐trans retinoic acid that binds directly to retinoic acid receptors [7]. HPR has demonstrated similar benefits to retinol and tretinoin in stimulating skin renewal and collagen synthesis, but with greater chemical stability and potentially lower irritation [8]. Combining HPR with retinol may allow synergistic effects: HPR could augment retinoid activity while permitting a reduced retinol concentration, thereby mitigating irritation while maintaining efficacy. However, the molecular mechanisms of HPR and its interaction with retinol remain incompletely understood. It is particularly unclear whether HPR activates the same biological pathways as retinol or if the combination yields additive rather than redundant effects.

Modern anti‐aging skincare formulations often integrate peptides and botanical antioxidants to target complementary aspects of skin aging. Specific peptides, such as tetradecyl aminobutyroyl valylaminobutyric urea trifluoroacetate and palmitoyl tripeptide‐5, have been shown to stimulate collagen production and improve skin firmness [9]. Silybin, the major active constituent of silymarin derived from *Silybum marianum* (milk thistle), exhibits potent antioxidant and anti‐inflammatory effects and can protect skin from UV‐induced oxidative stress [10, 11]. The inclusion of silybin in a retinoid‐based formulation could potentially enhance skin protection and reduce irritation during retinoid therapy [12]. Despite the popularity of such multi‐ingredient approaches, scientific evidence on their integrated effects remains limited, underscoring the importance of systematically evaluating multicomponent formulations.

In this study, we adopted an integrated approach combining mechanistic in vitro experiments and a clinical trial to develop and assess a novel low‐irritation anti‐aging formulation. Initially, a co‐culture model of human keratinocytes and dermal fibroblasts was established to simulate skin architecture, and we applied RNA sequencing (RNA‐seq) to characterize the effects of retinol and HPR at the transcriptomic level. We focused on pathways such as TGF‐β/Smad signaling, which are crucial for dermal extracellular matrix (ECM) maintenance [13]. Additionally, we evaluated the formulation with and without silybin in an ex vivo engineered skin tissue model, using immunohistochemical and immunofluorescent analyses of key proteins including transforming growth factor‐beta 1 (TGF‐β1), TGF‐β2, phosphorylated Smad2 (p‐Smad2), p‐Smad3, collagen types I, IV, and XVII, and elastin. Finally, a pivotal 8‐week clinical study was conducted using the optimized formulation containing retinol, HPR, peptides, and silybin. We assessed improvements in clinical signs such as wrinkles, texture, and elasticity, as well as overall tolerability. By combining mechanistic insight with clinical validation, our study provides a novel multi‐active anti‐aging serum and new perspectives on optimizing retinoid‐based skincare for populations sensitive to irritation.

## 2 Materials and Methods

### 2.1 Cells and Cell Culture

HaCaT keratinocytes and human dermal fibroblasts (BioCell Biotechnology, China) were cultured in DMEM (Gibco, UK) with 10% fetal bovine serum and 1× penicillin–streptomycin. HaCaT cells were seeded on transwell inserts, and fibroblasts were seeded in 6‐well plates. After overnight incubation at 37°C with 5% CO₂, HaCaT cells were treated with 10.875 μM of test samples for 8 h. Fibroblasts were concurrently exposed to UVA (30 J/cm^2^) and UVB (50 mJ/cm^2^). The experimental groups included: (1) retinol‐HPR combination, (2) retinol alone, (3) HPR alone, and (4) negative control. Inserts were placed above fibroblast cultures to establish a co‐culture system, which was incubated for 24 h before analysis.

### 2.2 Transcriptome and RT‐qPCR Analysis

RNA was extracted from fibroblasts using the MiniBEST kit (TaKaRa, Japan), followed by Illumina‐based RNA sequencing. Raw reads were filtered and aligned with Hisat2, and differential gene expression was analyzed using DESeq2. Gene Ontology (GO) and Kyoto Encyclopedia of Genes and Genomes (KEGG) enrichment were performed via clusterProfiler. RT‐qPCR was conducted on select genes, normalized to GAPDH. Primer sequences are available in Supporting Information (Table S1).

### 2.3 Skin Tissue Model and Immunostaining

Bioengineered skin tissues (BioCell Biotechnology, China) were irradiated (30 J/cm^2^ UVA and 50 mJ/cm^2^ UVB) and topically treated as per experimental group assignments:

DRDP: retinol + HPR + peptides;

DRDPS: DRDP + silybin;

PC1: Vitamin C (100 μg/mL) + Vitamin E (7 μg/mL) for collagen‐related markers;

PC2: TGF‐β1 (100 ng/mL) for TGF‐β/Smad pathway markers.

After treatment, tissues were fixed in 4% formaldehyde, embedded, sectioned, and stained for TGF‐β1, TGF‐β2, p‐Smad2, p‐Smad3, collagen I, IV, XVII, and elastin. Immunofluorescence and immunohistochemistry images were acquired using a Leica DM2500 microscope and analyzed via Image J. More details are available in Supporting Information, and group definitions and UV induction regimen are summarized in Table S2.

### 2.4 Human Efficacy Study Design

An 8‐week clinical study was conducted at SGS Testing Center, approved by an ethics committee. A comprehensive list of the serum's formula ingredients is detailed in Data S4. retinol and HPR levels were pre‐optimized by internal DOEs to balance efficacy and tolerability; exact percentages are proprietary. Inclusion and exclusion criteria are detailed in the Supporting Information (Table S3). All participants provided written informed consent. Thirty‐one women completed the study.

### 2.5 Instrumental Measurements

Wrinkles on the cheeks, canthus, and under the eyes were assessed with a Primos‐CR 3D roughness analyzer. Antera 3D evaluated wrinkles on the forehead, eyebrows, nasolabial fold, neck, and facial pores. VISIA CR camera images under cross‐polarized light were analyzed with Image‐Pro Plus (IPP) for L* and ITA° values. Skin elasticity, gloss, sebum levels, moisture, transepidermal water loss (TEWL), and texture were measured with Cutometer, Glossymeter, Sebumeter, Corneometer, Tewameter, and Visioscan, respectively.

### 2.6 Statistical Analysis

Data were analyzed using SPSS software. *t*‐test statistical analysis was used for comparison between skin tissue test groups and paired *t*‐test for clinical test comparisons between baseline and essence‐treated skin, with significance set at *p* < 0.05.

## 3 Results

### 3.1 Transcriptomic Analysis in co‐Culture Model

#### 3.1.1 Differential Gene Expression and Enrichment Analyses

Retinol, HPR, and their combination were applied to the co‐culture model, and global transcriptomic changes were visualized via heatmaps (Figure S1). A total of 2625 genes were differentially expressed (DEGs) in fibroblasts treated with the retinol‐HPR combination compared to retinol alone, while fewer DEGs were observed between the retinol‐HPR and HPR groups (Figure S2). This suggests that the biological effect of the combination was more similar to HPR than to retinol.

**FIGURE 1:**
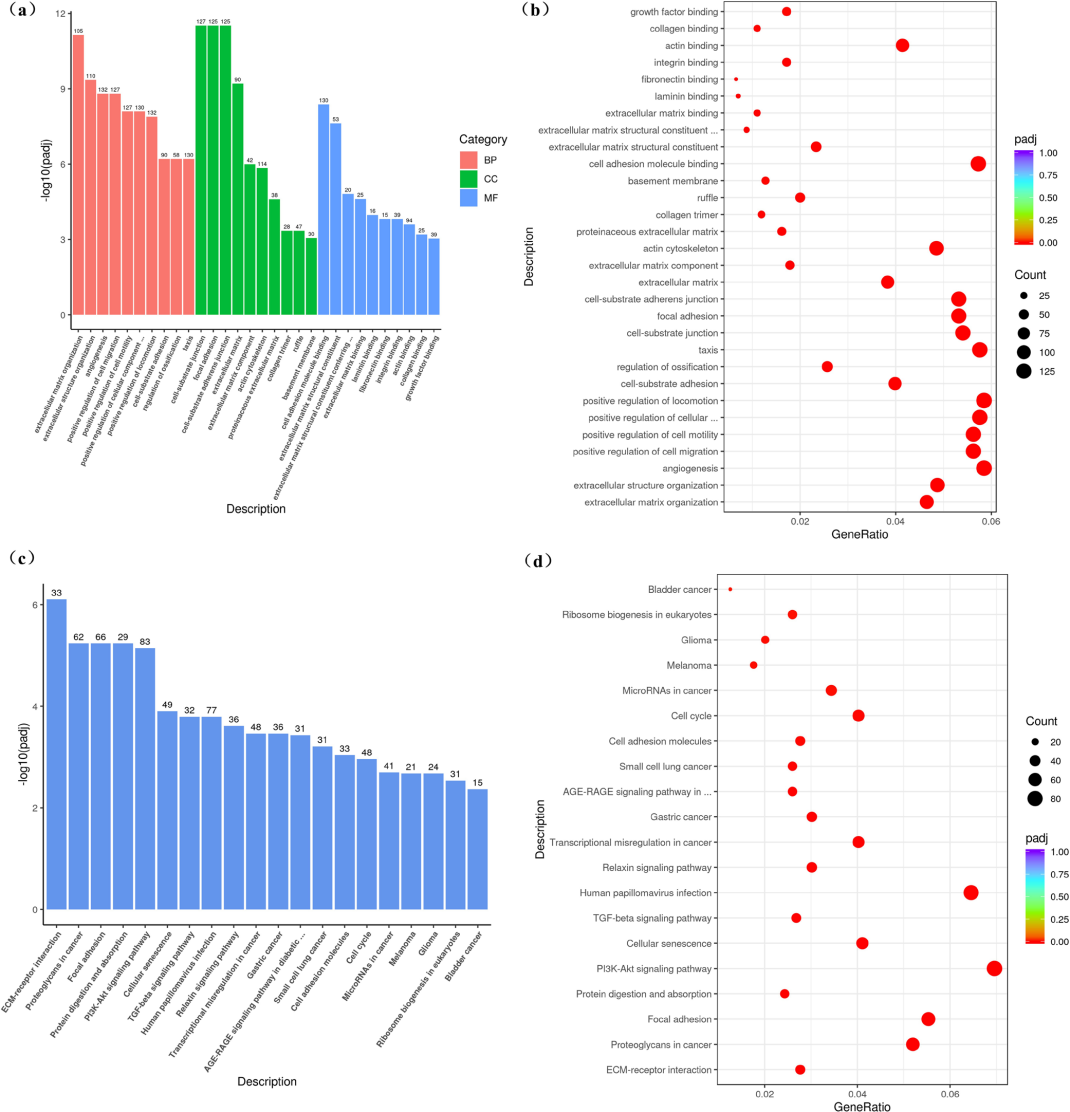
Differentially expressed gene statistics. (a, b) Bar chart and bubble diagram of GO enrichment analysis between Retinol‐HPR group and retinol group. (c, d) Bar chart and bubble diagram of KEGG enrichment analysis between retinol‐HPR group and retinol group.

GO enrichment analysis (Figure 1a,b) revealed that DEGs between the retinol‐HPR and retinol groups were significantly associated with ECM organization, extracellular structure organization, positive regulation of cell migration, and cell motility. KEGG pathway analysis (Figure 1c,d) showed significant enrichment in 69 pathways (*p* < 0.05), including ECM‐receptor interaction, focal adhesion, PI3K‐Akt signaling, cellular senescence, and the TGF‐β signaling pathway. These findings indicate that the retinol‐HPR combination primarily modulated ECM composition, cell adhesion, and migration, with distinct biological effects compared to retinol alone.

#### 3.1.2 Key Gene Expression Related to Photoaging

**FIGURE 2:**
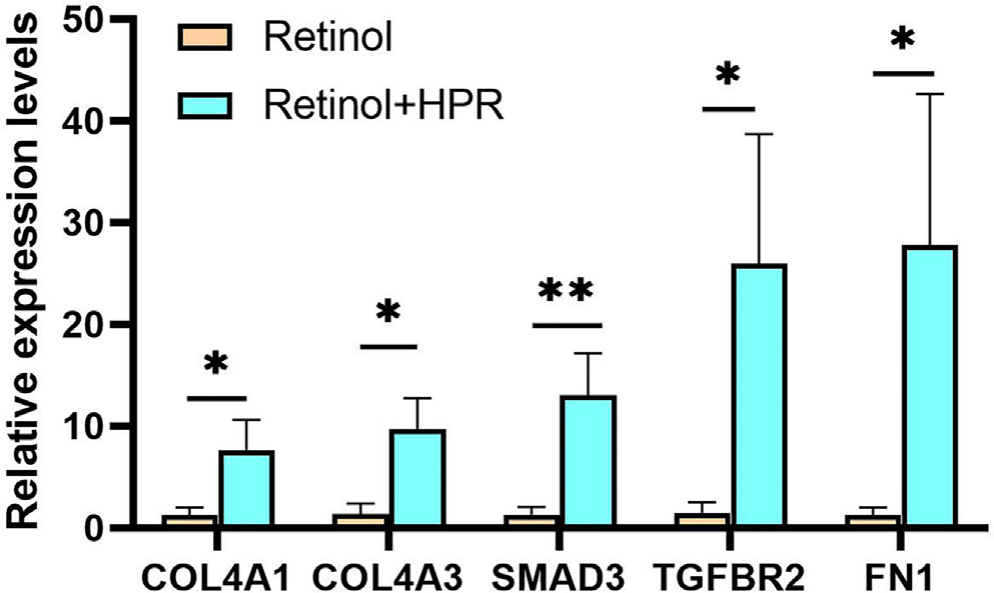
Validation of the expression for select genes. Statistical significance was assessed by Student's *t*‐test. Data are presented as mean ± SD (*p < 0.05, **p < 0.01).

To identify functionally relevant DEGs, we focused on pathways involved in cell proliferation, migration, and differentiation. Genes such as collagen type IV alpha 1 (COL4A1), collagen type IV alpha 2 (COL4A2), fibronectin 1 (FN1), SMAD family member 3 (SMAD3), and transforming growth factor beta receptor 2 (TGFBR2) showed significant upregulation in the retinol‐HPR group compared to the retinol group (Table 1), suggesting enhanced ECM remodeling. Notably, fibronectin‐related genes (FN1, integrin subunit beta 3 (ITGB3), ITGB5) and members of the TGF‐β pathway (TGFBR2, bone morphogenetic protein 6 (BMP6), growth differentiation factor 6 (GDF6), SMAD3) were upregulated, indicating increased fibroblast activity and ECM homeostasis [14, 15, 16, 17, 18]. These results were confirmed by RT‐qPCR, supporting the reliability of the RNA‐seq data (Figure 2).

**TABLE 1:** Gene expression changes in the retinol‐HPR group compared to the retinol group.

Gene	Retinol‐HPR vs. retinol^a^	*p*_adj_
*COL4A1*	+61.05%	1.03E‐14
*COL4A2*	+26.67%	2.27E‐04
*COL4A3*	+63.30%	4.67E‐01
*DCN*	+64.07%	2.59E‐15
*GDF6*	+121.44%	1.19E‐05
*FN1*	+43.11%	4.47E‐23
*ITGB3*	+38.18%	2.70E‐03
*ITGB5*	+15.63%	9.94E‐04
*MMP1*	−44.74%	2.74E‐47
*MMP3*	−34.32%	4.02E‐16
*LTBP1*	+40.29%	2.31E‐09
*TGFBR2*	+26.46%	1.01E‐08
*BMP6*	+447.65%	5.68E‐04
*SMAD3*	+22.42%	5.80E‐05

^a^ The gene expression changes of the retinol‐HPR group relative to the retinol group are presented as fold change—100%.

### 3.2 Silybin Enhances TGF‐β/Smad Signaling and ECM Protein Synthesis

**FIGURE 3:**
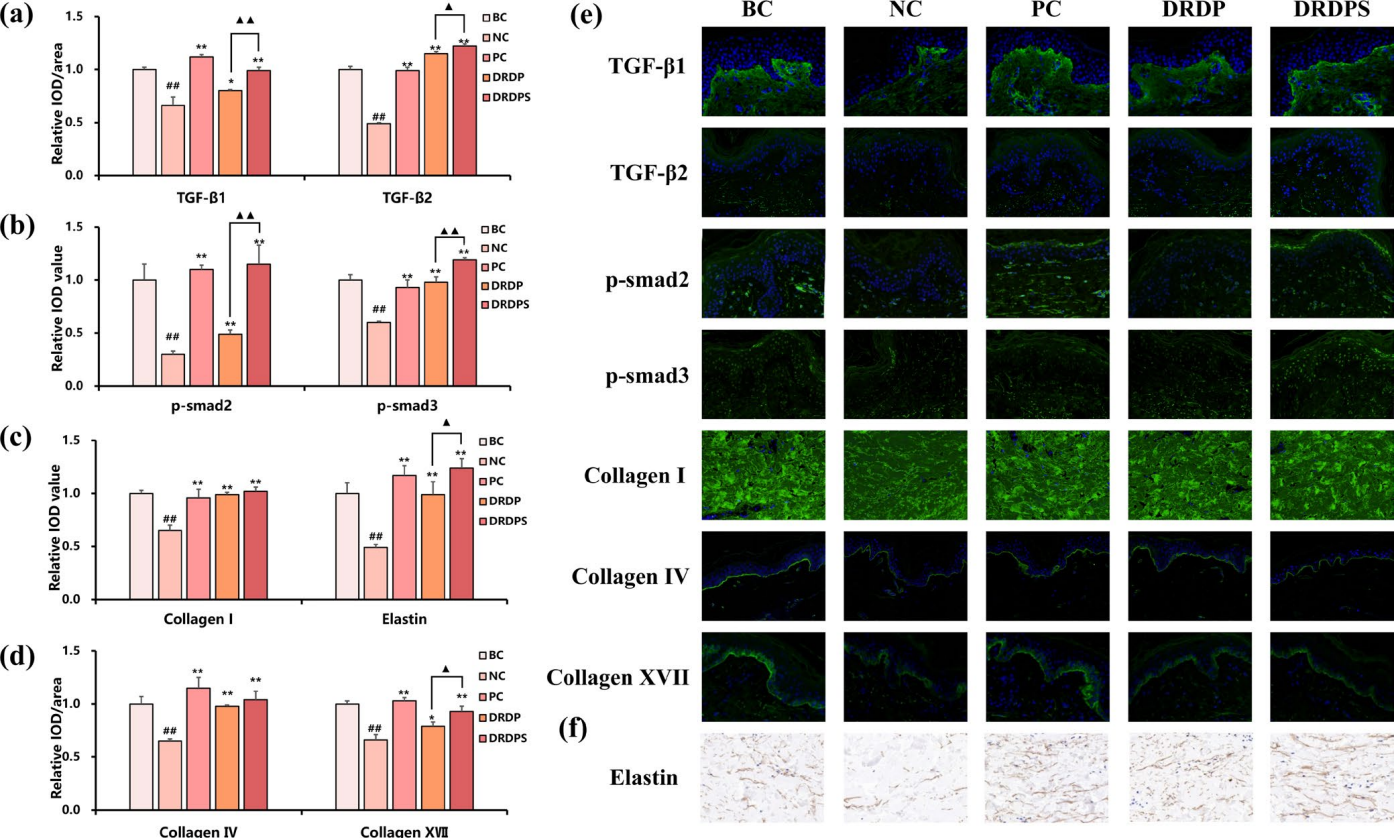
The key proteins related to the TGF‐β‐Smad signaling pathway and ECM. (a) Quantification of TGF‐β1 and TGF‐β2. (b) Quantification of p‐smad2 and p‐smad3. (c) Quantification of collagen I and elastin. (d) Quantification of collagen IV and collagen XVII. Data are presented as Relative IOD/Area Mean. IOD stands for integrated optical density, which is a measure of the total fluorescence intensity in a specific area. Data are presented as mean ± SD (^##^ *p* < 0.01, vs. BC; *p < 0.05, **p < 0.01, vs. NC; ^▲^ *p* < 0.05, ^▲▲^ *p* < 0.01, vs. DRDP). (e) Immunofluorescence images of TGF‐β1, TGF‐β2, p‐smad2, p‐smad3, collagen I, collagen IV and collagen XVII. The intensity of green fluorescence indicates the protein content. (f) Immunohistochemistry images of elastin. The intensity of brown color indicates the elastin.

Given the gene expression data suggesting that the retinol+HPR combination stimulates TGF‐β/Smad pathways and collagen production, we investigated whether adding silybin could further boost these pro‐collagen effects. In an ex vivo skin model, we compared the retinol + HPR + peptides base formula (DRDP) with the same formula supplemented with silybin (DRDPS). The TGF‐β/Smad signaling pathway plays a central role in maintaining dermal ECM by regulating collagen and fibronectin synthesis [19, 20]. Immunostaining results are quantified in Figure 3a–d. The DRDP treatment significantly increased protein levels of TGF‐β1 and TGF‐β2 in the skin equivalent compared to untreated control (negative control, NC). With the addition of silybin (DRDPS), TGF‐β1 protein levels rose 23.8% further, and TGF‐β2 by 6.1% beyond the levels induced by DRDP alone (Figure 3a). Similarly, DRDP elevated phosphorylated Smad2 and Smad3 (downstream effectors of TGF‐β) relative to NC; notably, DRDPS further boosted p‐Smad2 by 134.7% and p‐Smad3 by 21.4% over the DRDP group (Figure 3b). These results indicate that silybin enhances the activation of the TGF‐β/Smad pathway triggered by the retinol + HPR + peptide combination.

Consistent with the upregulation of TGF‐β signaling, DRDP treatment markedly increased the expression of ECM proteins. Collagen I (the major dermal collagen) was elevated in the DRDP group, along with collagen IV (a basement membrane component) and collagen XVII (a hemidesmosomal collagen important for dermal–epidermal adhesion). With silybin (DRDPS), collagen XVII levels increased an additional 17.7% compared to DRDP alone, indicating a further enhancement of basement membrane components (Figure 3d). Elastin, which provides elastic recoil to skin, was also significantly upregulated by DRDP. Importantly, the DRDPS formulation led to a 25.3% higher elastin content than DRDP (Figure 3c), suggesting that silybin helps preserve or stimulate elastin synthesis as well. Representative immunofluorescence images (Figure 3e) show the intensity of green fluorescence corresponding to each protein target, illustrating qualitatively that DRDPS‐treated skin had the strongest staining for key markers like p‐Smad2/3 and collagens. Immunohistochemical detection of elastin (Figure 3f, brown staining) similarly shows more intense elastin in DRDPS vs. DRDP. Overall, these ex vivo results demonstrate that adding silybin to the retinol–HPR–peptide formulation amplifies its pro‐ECM effects, likely by both promoting TGF‐β/Smad signaling and providing antioxidant protection to the skin matrix.

### 3.3 8‐Week Clinical Performance of the Optimized Retinoid Serum

#### 3.3.1 Wrinkle Reduction

**FIGURE 4:**
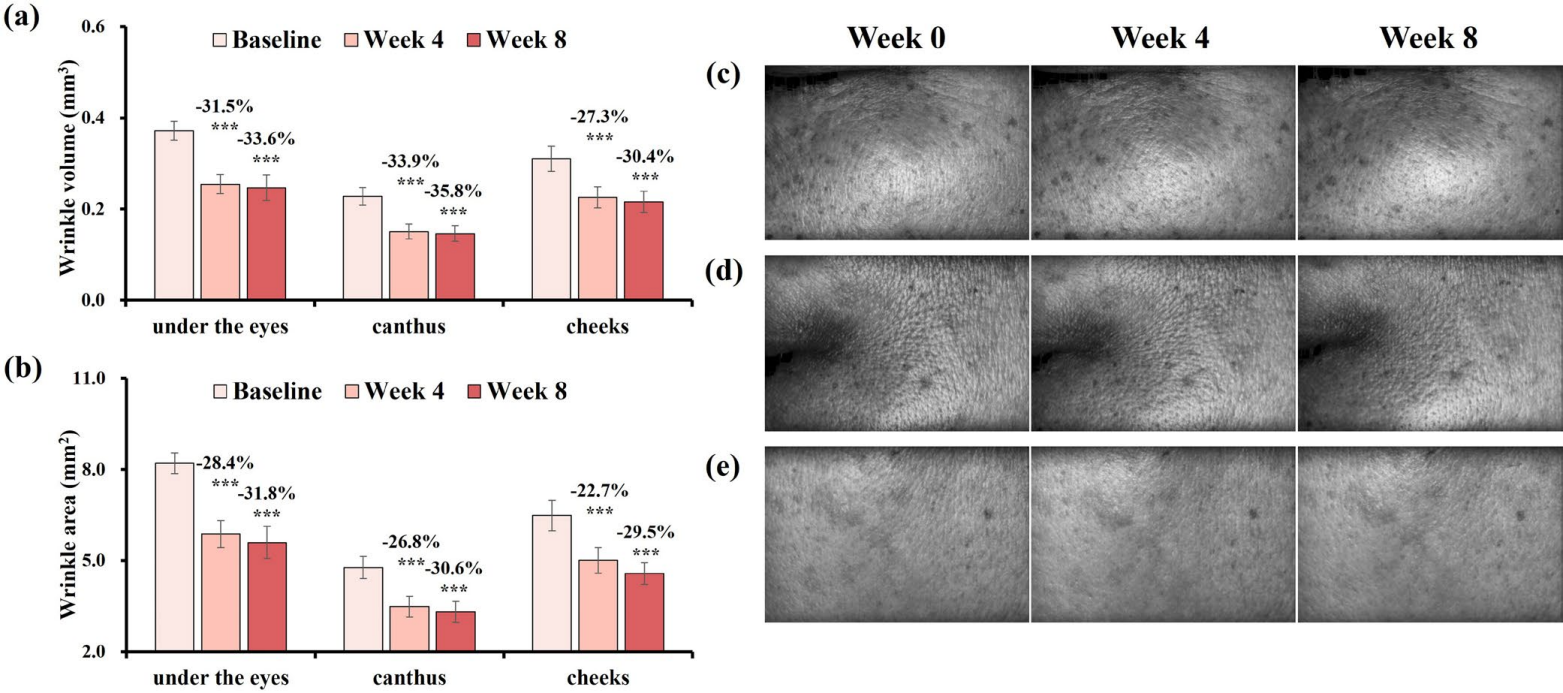
Changes in wrinkle volume and area on the cheeks, canthus, and under‐eye areas after 8 weeks, with Primos‐CR images. (a) Wrinkle volume. (b) Wrinkle area. Change rate (%) at week 4/8 = (week 4/8 value—baseline value)/baseline value was used to assess parameter improvement. Data are presented as mean ± SD (***p < 0.001, vs. Baseline). (c) Under‐eye (subject 27, female, age: 48 years old). (d) Canthus (subject 16, female, age: 39 years old). (e) Cheeks (subject 21, female, age: 60 years old).

**FIGURE 5:**
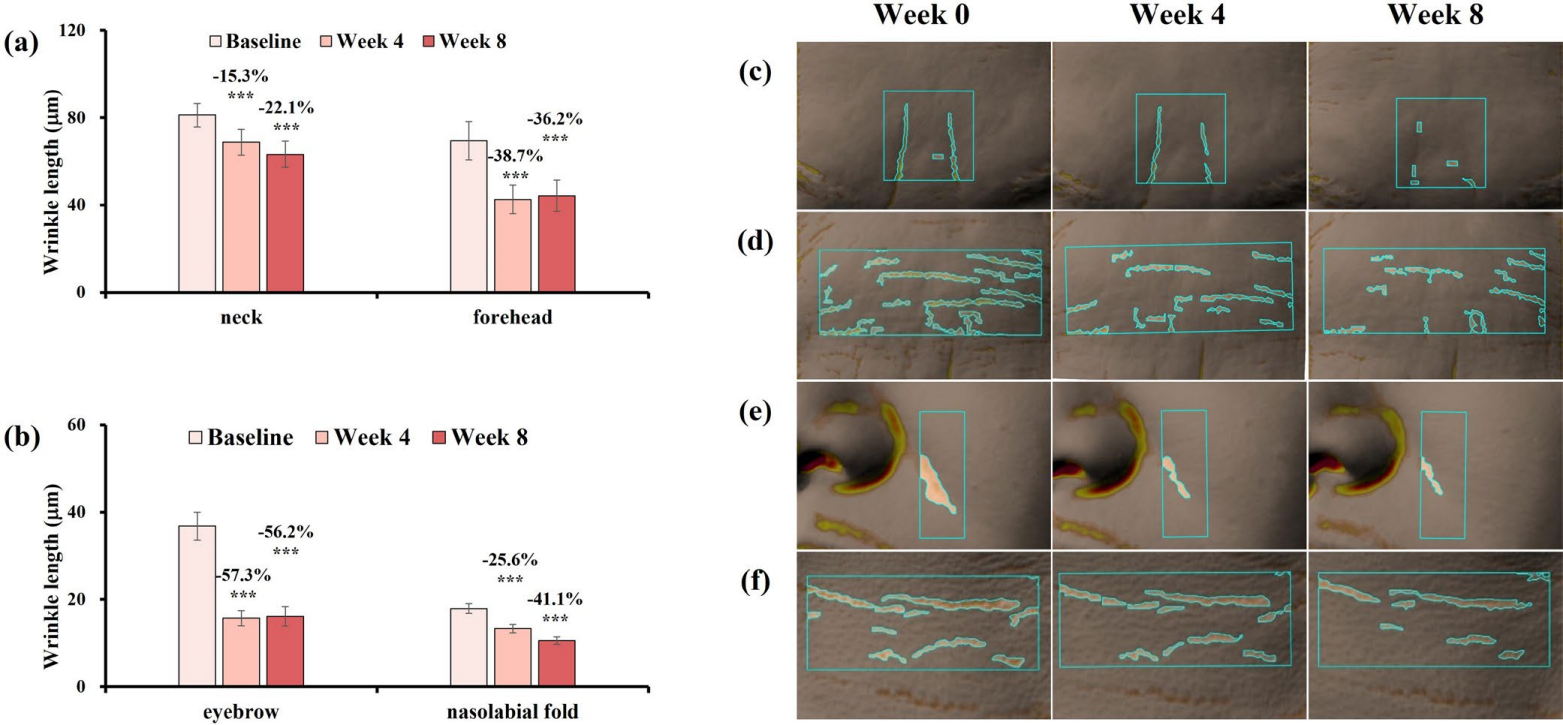
Changes in wrinkle length on the neck, forehead, eyebrows, and nasolabial fold after 8 weeks with Antera 3D images. (a) Neck and forehead. (b) Eyebrows and nasolabial fold. Change rate (%) at week 4/8 = (week 4/8 value—baseline value)/baseline value was used to assess parameter improvement. Data are presented as mean ± SD (***p < 0.001, vs. Baseline). (c) Eyebrows (subject 21, female, age: 60 years old). (d) Forehead (subject 24, female, age: 52 years old). (e) Nasolabial fold (Subject 12, female, age: 38 years old). (f) Neck (subject 27, female, age: 48 years old).

After 8 weeks of treatment, participants exhibited significant reductions in wrinkle volume and area across the cheeks, canthus, and under‐eye regions, as well as wrinkle length on the forehead, nasolabial folds, neck, and brows (Figures 4 and 5). These improvements were supported by 3D imaging, indicating broad efficacy across facial zones.

#### 3.3.2 Elasticity and Firmness

**FIGURE 6:**
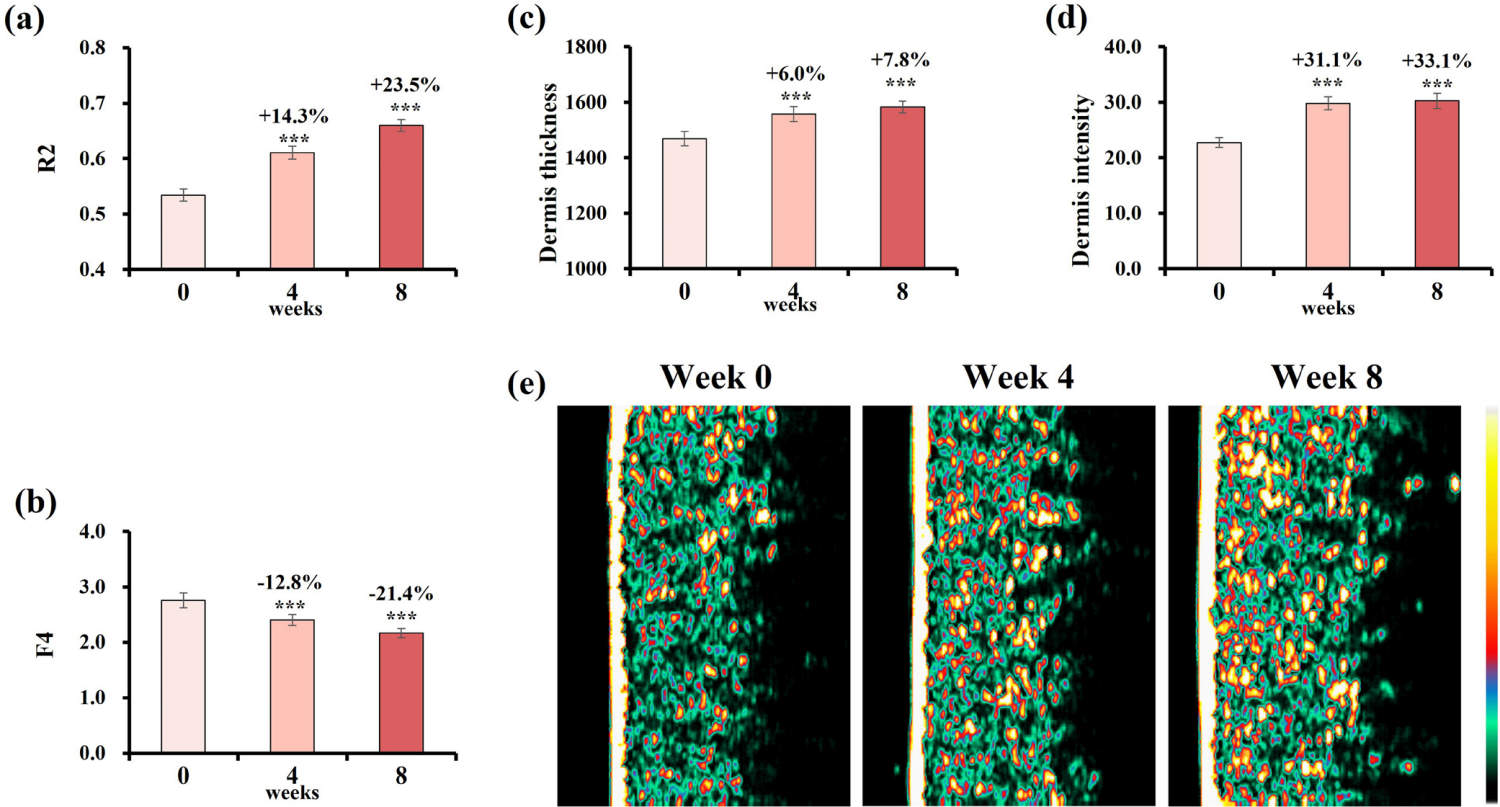
Changes in skin elasticity, firmness, thickness and intensity over 8 weeks. (a) R2 value. (b) F4 value. (c) Thickness. (d) Intensity. Change rate (%) at week 4/8 = (week 4/8 value—baseline value)/baseline value was used to assess parameter improvement. Data are presented as mean ± SD (***p < 0.001, vs. baseline). (e) Representative Dermalab ultrasound images (Subject 12, female, age: 38 years old).

Skin elasticity improved, as reflected by a 23.5% increase in R2 values and a 21.4% decrease in F4 values (Figure 6a,b). Ultrasound analysis further confirmed improvements in dermal thickness and intensity (Figure 6c–e), suggesting enhanced structural integrity of the dermis.

#### 3.3.3 Texture and Pore Refinement

**FIGURE 7:**
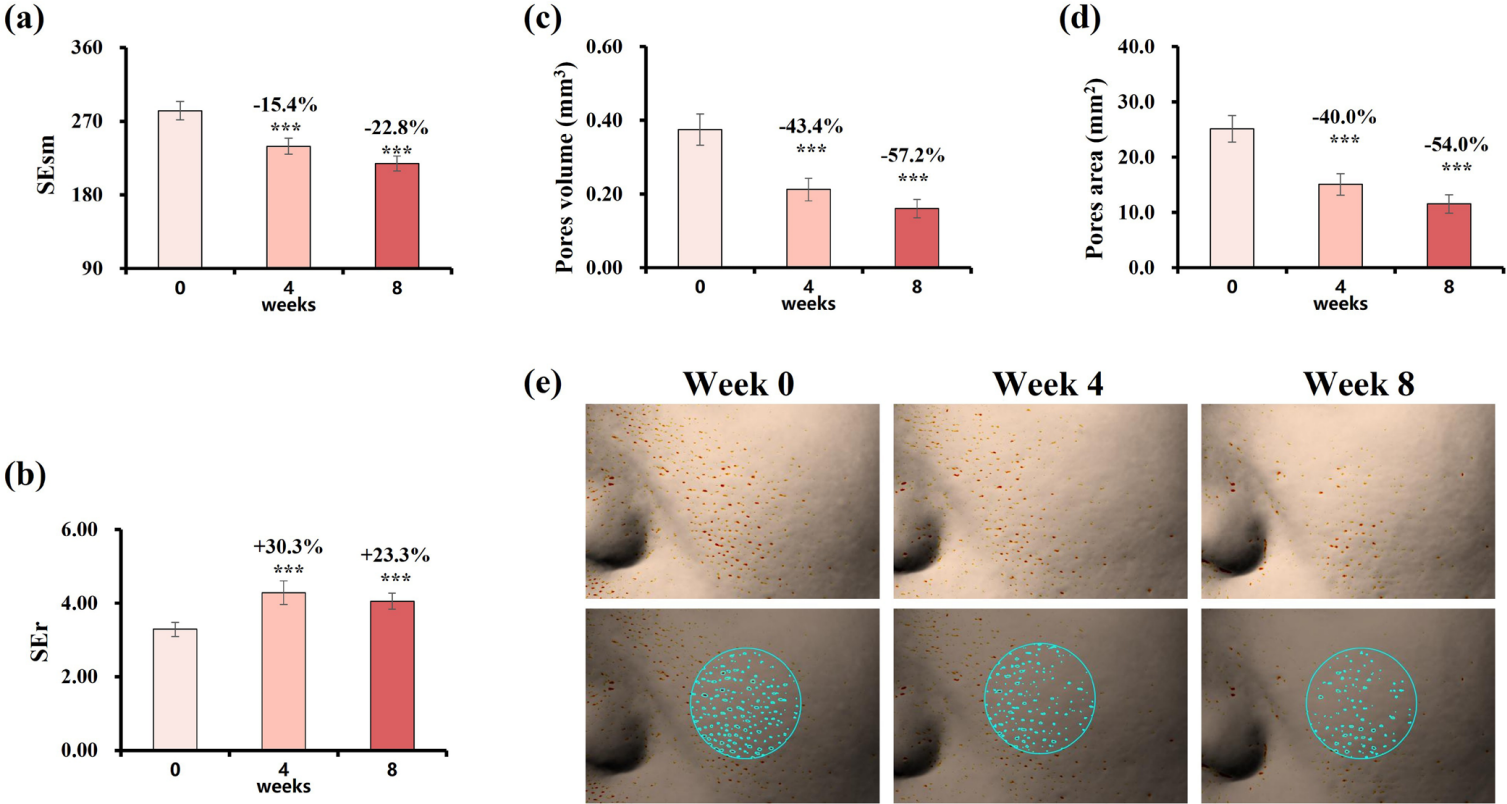
Changes in skin roughness, smoothness, and pore characteristics over 8 weeks. (a) SEr value. (b) SEsm value. (c) Pore volume. (d) Pore area. Change rate (%) at week 4/8 = (week 4/8 value—baseline value)/baseline value was used to assess parameter improvement. Data are presented as mean ± SD (***p < 0.001, vs. baseline). (e) Representative Antera 3D images of pore improvement (Subject 04, female, age: 36 years old).

After 8 weeks, skin roughness (SEr) increased by 23.3%, while smoothness (SEsm) decreased by 22.8% (Figure 7a,b). Pore volume and area decreased by 57.2% and 54.0%, respectively (Figure 7c,d), with representative images demonstrating visible pore refinement (Figure 7e).

#### 3.3.4 Hydration, Barrier Function, and Complexion

**FIGURE 8:**
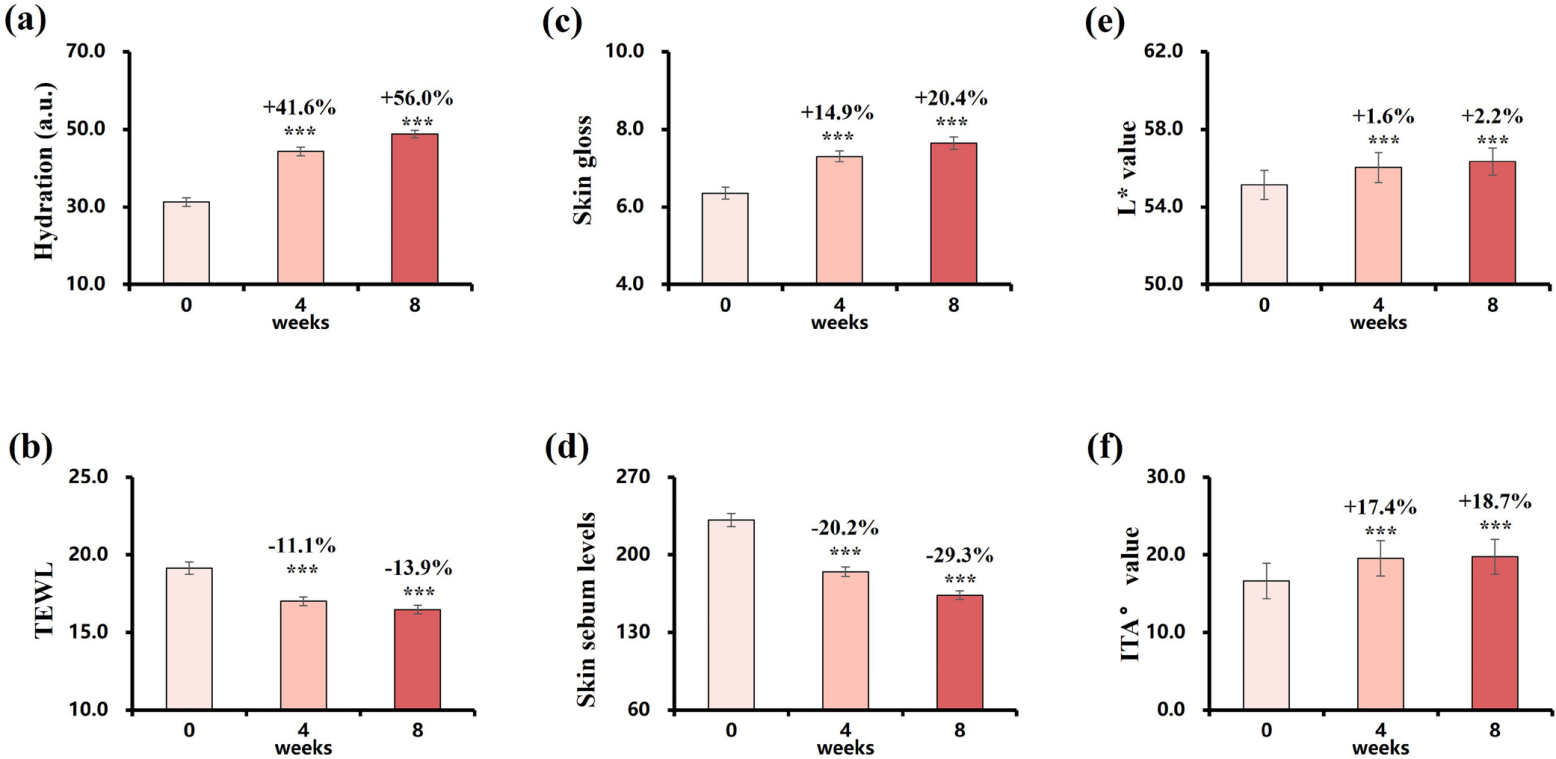
Changes in skin hydration, TEWL, gloss, sebum levels, and color over 8 weeks. (a) Hydration. (b) TEWL. (c) Gloss. (d) Sebum levels. (e) L* value. (f) ITA° value. Change rate (%) at week 4/8 = (week 4/8 value—baseline value)/baseline value was used to assess parameter improvement. Data are presented as mean ± SD (***p < 0.001, vs. baseline).

Stratum corneum hydration increased by 56.0% (Figure 8a), accompanied by a significant reduction in TEWL (Figure 8b), indicating enhanced barrier function. Skin gloss improved at both weeks 4 and 8 (Figure 8c), while sebum levels on the forehead gradually declined (Figure 8d). Skin tone parameters also improved: L* values increased by 2.2%, and ITA° values rose by 18.7% (Figure 8e,f), reflecting a brighter complexion.

## 4 Discussion

Topical retinoids remain foundational in anti‐aging therapy due to their proven ability to improve photodamaged skin, but their use is often limited by irritation—particularly in individuals with more sensitive skin types, such as many Asians [3, 4, 5, 6]. In this work, we developed and comprehensively evaluated a multicomponent serum combining retinol, HPR, peptides, and silybin. Through an integration of transcriptomic analysis, ex vivo skin modeling, and a clinical trial, we demonstrated that this formulation can significantly improve signs of mild photoaging while maintaining excellent tolerability in middle‐aged Chinese women.

At the molecular level, our co‐culture transcriptomic analysis revealed that retinol and HPR together modulate both overlapping and unique biological pathways compared to each retinoid alone. Notably, the combination more closely resembled HPR's gene expression profile than retinol's, suggesting that HPR may dominate certain retinoid responses when present. Gene enrichment results highlighted that the retinol+HPR treatment upregulated pathways related to the ECM and cell–matrix interactions (e.g., ECM‐receptor interaction, focal adhesion) and downregulated matrix‐degrading enzymes [21, 22]. In particular, we observed enhanced expression of multiple collagen genes (COL4A1, COL4A2) and fibronectin (FN1), alongside increased SMAD3 and TGFBR2, which are central to TGF‐β/Smad signaling. These data imply that HPR, when combined with retinol, augments dermal matrix remodeling rather than simply duplicating retinol's effect. Importantly, genes like FN1 and integrins ITGB3/5—critical players in fibroblast migration and wound healing—were elevated by the combination, pointing to a more robust fibroblastic repair and ECM production response [23, 24]. Such a response is desirable for anti‐aging, as photoaged skin often suffers from fragmented collagen fibrils and weakened dermal support structure [18, 20].

These transcriptional changes translated into concrete protein‐level outcomes. In the ex vivo skin model, the retinol + HPR (with peptides) treatment led to greater deposition of collagen types I, IV, XVII, and elastin, along with increased activation of p‐Smad2 and p‐Smad3, compared to controls. This indicates that the combination activates the TGF‐β‐mediated pathway known to drive collagen synthesis and dermal matrix assembly [25, 26, 27]. The addition of peptides in the formulation likely contributed to this effect. Notably, palmitoyl tripeptide‐5 can stimulate the release of active TGF‐β from its latent complex. In the nonactivated state, TGF‐β is a precursor complex bound to LAP protein. Palmitoyl tripeptide‐5 can release TGF‐β from the LAP‐TGF‐β complex, further regulating downstream signaling [28]. In addition, tetradecyl aminobutyroyl valylaminobutyric urea trifluoroacetate has been shown in earlier research to promote collagen production, supporting its inclusion as a complementary ingredient. Although we did not test a peptide‐free retinol + HPR formulation in the skin model, the observed upregulation of TGF‐β1/TGF‐β2 and Smad phosphorylation in the DRDP group aligns with the known mechanisms of peptides enhancing ECM remodeling [29]. Thus, our results support the view that incorporating peptides amplifies the pro‐collagen effects of retinoids, making the overall formulation more efficacious in rebuilding dermal structure.

Silybin acted as a supportive adjunct in our formula: compared with DRDP (retinol + HPR + peptides), DRDPS (adding silybin) showed further increases in p‐Smad2/3 and incremental gains in collagen (notably collagen XVII) and elastin in the ex vivo model. Beyond its well‐documented antioxidant/anti‐inflammatory properties—likely relevant to barrier comfort and tolerance—we examined whether silybin might influence retinoid homeostasis. In UV‐challenged human skin, our exploratory analysis (Data S7) quantified CYP26B1 immunoreactivity (IOD/Area) and found that retinol alone and silybin alone each showed lower CYP26B1 signal vs. controls, whereas the combination yielded the largest reduction (Figure S3). Because this readout reflects protein levels rather than catalytic turnover, and no benchmark inhibitor (e.g., talarozole/R115866) or direct activity assay was employed, we cannot infer CYP26B1 enzymatic inhibition nor causality for sustained retinoid signaling. Nevertheless, together with literature on CYP26‐mediated RA clearance [30], these observations are consistent with a scenario in which CYP26B1 modulation may contribute to the overall retinoid‐responsive ECM improvements. Definitive testing will require enzyme activity assays, time‐course expression/turnover studies, and pathway perturbation.

Encouragingly, the in vitro and ex vivo findings translated into robust clinical improvements. After 8 weeks of application, the multi‐ingredient serum produced visible and measured benefits: wrinkles were reduced in depth and length across multiple facial areas, skin elasticity and firmness increased, and complexion became brighter and more even. At the same time, reported irritation was minimal. This outcome is noteworthy given that retinol's efficacy is often accompanied by irritation in many users [3]. In our study, no participant experienced more than mild, transient dryness or redness, which is a marked contrast to the significant peeling and erythema frequently seen with classical retinoid regimens of comparable strength. The favorable tolerability of our serum suggests that including HPR as a partial retinoid substitute successfully mitigated the typical retinol‐associated side effects without sacrificing efficacy. HPR's direct receptor activation and lower irritation profile [7, 8] likely allowed us to maintain strong dermal remodeling activity at a lower effective retinol concentration. In effect, the combination broadened the therapeutic window of retinoid treatment—an outcome that is particularly valuable for populations prone to sensitivity. These results echo the hypothesis that by using HPR to shoulder some of the retinoid activity, one can reduce the burden on retinol itself, thereby reducing irritation while still achieving significant skin improvements.

Our findings also underscore the value of a multi‐pathway, multi‐ingredient strategy in anti‐aging skincare. Rather than relying on a single active, the formulation targets skin aging on several fronts: retinoids (retinol+HPR) to stimulate cell turnover and collagen, peptides to further boost collagen and signal repair, and silybin to protect against oxidative stress and enhance tolerance. The clinically observed increases in hydration and barrier function can also be attributed to this holistic approach—retinoids encourage exfoliation and renewal, peptides support barrier proteins, and silybin reduces irritation that could impair the barrier. The net result was smoother, firmer, and more radiant skin with minimal downtime. To our knowledge, this study is among the first to integrate a transcriptomic analysis with a clinical trial for a retinol+HPR combination product and to demonstrate synergistic benefits of adding peptides and an antioxidant in a sensitive‐skin cohort.

There are some limitations to note. First, while our data strongly suggest that the TGF‐β/Smad pathway is a major mediator of the observed effects, we did not employ specific pathway inhibitors (such as a TGF‐β receptor blocker or Smad knockdown) to conclusively prove causality in the mechanistic studies. Future research could utilize such inhibitors to confirm that the pro‐collagen effects are indeed Smad‐dependent. Second, our clinical study was an open‐label, single‐arm trial without a comparator. Although the improvements were significant and consistent with known retinoid effects, a randomized controlled trial against a retinol‐only product or a placebo would strengthen the evidence for the added benefits of HPR, peptides, and silybin. Lastly, we chose not to disclose the exact concentration of key actives in the formula due to proprietary considerations. The approximate concentration ranges of these actives are provided in Supporting Information. The concentration was guided by extensive internal testing to balance efficacy and tolerability. Despite not detailing those optimization studies here, the excellent clinical outcomes and low irritation index speak to the success of that concentration selection.

In conclusion, this study highlights the development of a potent yet low‐irritation anti‐aging serum. By integrating retinol with HPR, we harnessed synergistic retinoid activity that upregulated pro‐collagen pathways more than retinol alone while substantially improving tolerability. The addition of collagen‐stimulating peptides and the antioxidant silybin further enhanced skin improvements, creating a well‐rounded formulation that targets multiple aging mechanisms. Clinically, the serum significantly improved wrinkles, firmness, hydration, and pigmentation in photoaged Chinese women, with minimal side effects. These findings support a rational, multi‐faceted approach to optimizing retinoid therapies—one that addresses efficacy and safety in tandem. The in vitro–to–in vivo workflow used here provides a model for future skincare product development, wherein mechanistic insights guide formulation design and end‐result efficacy is validated in the target user population. In conclusion, this study demonstrates that the multi‐active serum combining retinol, HPR, peptides, and silybin significantly improved mild photoaged facial skin in Chinese women, with minimal irritation. While the clinical trial was limited to a population with mild photoaging, the favorable tolerability profile indicates that the formulation may be potentially suitable for individuals with sensitivity‐prone or easily irritated skin. Further studies in broader and more diverse populations are warranted to confirm this potential. Overall, our findings support a rational, multi‐pathway approach to optimizing retinoid‐based skincare for both efficacy and tolerability.

## Author Contributions

**Yixin Shen:** conceptualization, methodology, software, validation, formal analysis, investigation, data curation, writing – original draft preparation, writing – review and editing, visualization. **Muyang Shi:** methodology, software, validation, formal analysis, investigation, data curation, writing – original draft preparation, visualization. **Ying Ye:** conceptualization, methodology, investigation, writing – review and editing, supervision, project administration. **Chenlan Xu:** methodology, software, validation, formal analysis, investigation, data curation, writing – original draft preparation, writing – review and editing, visualization. **Xiao Feng, Tianyu Bi, and Yuyan Chen:** investigation. **Jiefang Huang:** investigation, project administration. **Yanan Li:** conceptualization, methodology, resources, writing – review and editing, supervision, funding acquisition. **Peiwen Sun:** methodology, resources, writing – review and editing, supervision, funding acquisition.

## Funding

This work was supported by Proya Cosmetics Co. Ltd.

## Ethics Statement

The study was conducted in accordance with the Declaration of Helsinki and approved by SGS Clinical Research Ethics Committee (Approval No. HZCPCH24000001). The participants provided written informed consent for the use of their clinical images/data for publication. Access to biopsy material was in accordance with Chinese law and satisfied the requirements of the local Ethics Committee.

## Conflicts of Interest

The authors declare no conflicts of interest.

## Supporting information


**Table S1:** Primer sequences used for RT‐qPCR validation. Forward and reverse sequences (5′‐3′) for COL4A1, COL4A3, FN1, SMAD3, and TGFBR2, GAPDH served as the housekeeping gene.
**Table S2:** Experimental groups and UV induction regimen for the ex vivo skin model.
**Table S3:** Inclusion and exclusion criteria for the human efficacy study.
**Figure S1:** Heat map of DEGs.
**Figure S2:** Volcano plots of DEGs in retinol‐HPR group vs. retinol group (a) and retinol‐HPR group vs. HPR group (b). The blue dashed line indicates the threshold line for the differentially expressed gene screening criteria.
**Figure S3:** CYP26B1 levels were assessed by Immunofluorescence. Data are presented as Relative IOD/Area Mean. IOD stands for integrated optical density, which is a measure of the total fluorescence intensity in a specific area. Data are presented as mean ± SD (^##^ *p* < 0.01, vs. BC; *p < 0.05, **p < 0.01, vs. NC).

## Data Availability

The datasets used and/or analyzed during the current study are available from the corresponding author on reasonable request.
