# Supplementary material for: An Innovative Serum With Retinol, Hydroxypinacolone Retinoate, Peptides, and Silybin Improves Mild Photoaged Facial Skin in Middle‐Aged Chinese Women
Source: J Cosmet Dermatol. 2025 Dec 25;25(1):e70627. doi: 10.1111/jocd.70627 (PMC12741608; doi:10.1111/jocd.70627)
Supplement: Supplementary file 1 — Table S1: Primer sequences used for RT‐qPCR validation. Forward and reverse sequences (5′‐3′) for COL4A1, COL4A3, FN1, SMAD3, and TGFBR2, GAPDH served as the housekeeping gene. Table S2: Experimental groups and UV induction regimen for the ex vivo skin model. Table S3: Inclusion and exclusion criteria for the human efficacy study. Figure S1: Heat map of DEGs. Figure S2: Volcano plots of DEGs in retinol‐HPR group vs. retinol group (a) and retinol‐HPR group vs. HPR group (b). The blue dashed line indicates the threshold line for the differentially expressed gene screening criteria. Figure S3: CYP26B1 levels were assessed by Immunofluorescence. Data are presented as Relative IOD/Area Mean. IOD stands for integrated optical density, which is a measure of the total fluorescence intensity in a specific area. Data are presented as mean ± SD (## p < 0.01, vs. BC; *p < 0.05, **p < 0.01, vs. NC). [file JOCD-25-e70627-s001.docx]

**An Innovative Serum with Retinol, Hydroxypinacolone Retinoate, Peptides, and Silybin Improves Mild Photoaged Facial Skin in Middle-Aged Chinese Women**

**Data S1：Detailed RNA Isolation and Transcriptome Sequencing**

Total RNA was extracted from fibroblasts using the MiniBEST Universal RNA Extraction Kit (TaKaRa, Japan). cDNA libraries were constructed following standard Illumina protocols. Library quality was assessed with an Agilent Bioanalyzer 2100 system. Cluster generation was performed using a cBot Cluster Generation System with a TruSeq PE Cluster Kit v3-cBot-HS (Illumina). Sequencing was conducted on an Illumina NovaSeq platform to generate 150 bp paired-end reads.

Raw reads were processed using fastp to obtain clean data. Reference genome alignment was performed using Hisat2 v2.0.5, and gene-level counts were generated with FeatureCounts v1.5.0-p3. Differential gene expression analysis was conducted with the DESeq2 R package (v1.20.0), considering adjusted p-values ≤ 0.05 as statistically significant. Gene Ontology (GO) and Kyoto Encyclopedia of Genes and Genomes (KEGG) enrichment analyses were performed using the clusterProfiler R package.

**Data S2：Detailed Quantitative Reverse Transcription PCR (RT-qPCR)**

cDNA synthesis was carried out using the PrimeScript II 1st Strand cDNA Synthesis Kit (TaKaRa, Japan), following the manufacturer’s instructions. RT-qPCR was performed with TB Green® Fast qPCR Mix (TaKaRa, Japan) , with the primer sequences shown in Table S1. Gene expression was quantified using the 2^−ΔΔCT method, with GAPDH serving as the housekeeping gene.

**Supplementary TABLE S1.** Primer sequences used for RT-qPCR validation. Forward and reverse sequences (5′ -3′) for COL4A1, COL4A3, FN1, SMAD3, and TGFBR2, GAPDH served as the housekeeping gene.

| Gene | Primer Sequence (Forward/Reverse 5′ -3′) |
| --- | --- |
| *COL4A1* | Forward: GTGTTGCAGGAGTGCCATTG  Reverse: ACTACAAAAGCTAAGAAGCTGGT |
| *COL4A3* | Forward: GCCTGATGGTGAACCAGGAA  Reverse: CCAGGACAACCCAGTGATCC |
| *FN1* | Forward: AAGCTCAAGTGGTCCTGTCG  Reverse: CCAAGGTTTCTGGGTGGGAT |
| *SMAD3* | Forward: GGGGGTTGGACTTTCCTTCC  Reverse: GACTCCAAGTGGCAGCAGAA |
| *TGFBR2* | Forward: GTAGCTCTGATGAGTGCAATGAC  Reverse: CAGATATGGCAACTCCCAGTG |

**Data S3：Detailed Skin Tissue Model and Immunostaining**

**Skin Tissue Treatment**

Skin tissues (BioCell Biotechnology, China) were washed and sectioned into 24 ± 2 mm² pieces, epidermis side up, and placed into culture molds. The molds were transferred into 6-well plates and incubated at 37°C with 5% CO₂. After two days, tissues were irradiated and treated according to Table S2 protocols: UVA (30 J·cm^-2^) and UVB (50 mJ·cm^-2^) irradiation for four consecutive days, followed by topical application of test samples.

The experimental groups included:

• DRDP: retinol + HPR combined with peptides (tetradecyl aminobutyryl valamidobutyric urea trifluoroacetate and palmitoyl tripeptide-5),

• DRDPS: DRDP plus silybin.

Positive controls:

• PC1: Vitamin C (100 μg/mL) + Vitamin E (7 μg/mL) for collagen-related markers,

• PC2: TGF-β1 (100 ng/mL) for TGF-β/Smad pathway markers.

Following irradiation, tissues were cultured for an additional 3 days with drug administration but no further irradiation. After treatment, tissues were gently cleaned with sterile PBS and wiped with sterile cotton swabs.

**Supplementary Table S2.** Experimental groups and UV induction regimen for the ex vivo skin model.

| Groups | Samples | Induction condition |
| --- | --- | --- |
| Blank control（BC） | / | / |
| Negative control（NC） | / | 30 J·cm^-2^ UVA+50 mJ·cm^-2^ UVB |
| Positive control（PC1） | VC+VE |  |
| Positive control（PC2） | TGF-β1 |  |
| Experimental group | DRDP |  |
|  | DRDPS |  |

*****Group definitions: BC, blank control; NC, negative control (30 J·cm^-2^ UVA+50 mJ·cm^-2^ UVB); PC1, vitamin C (100 μg/mL) + vitamin E (7 μg/mL); PC2, TGF-β1 (100 ng/mL); DRDP, retinol + HPR + peptides; DRDPS, DRDP + silybin.

**Immunofluorescence Assay**

Skin tissues were fixed with 4% formaldehyde (Biosharp, Canada), dehydrated, embedded in paraffin, and sectioned. Sections were stained with antibodies targeting TGF-β1, TGF-β2 (Abcam, UK), p-Smad2, p-Smad3 (CST, USA), collagen I (CST, USA), collagen IV (Abcam, UK), and collagen XVII (Abcam, UK). Fluorescence images were captured with a Leica DM2500 microscope and analysed using ImageJ software.

**Immunohistochemistry Assay**

For elastin evaluation, tissues were similarly fixed, embedded, and stained with elastin-specific antibodies (Abcam, UK). Images were obtained using a Leica DM2500 fluorescence microscope and quantified with ImageJ.

**Data S4：The Ingredient List of Serum Formula**

The ingredients of the serum used in this study are as follows: water, dimethicone, acetyl glucosamine, glycerin, isotridecyl isononanoate, panthenol, ammonium acryloyldimethy ltaurate/VP copolymer, dimethyl isosorbide, 1,2-hexanediol, butylene glycol, hydroxyacetophenone, caprylic/capric triglyceride, hydroxypropyl cyclodextrin, hydrogenated lecithin, hexyldecanol, pentylene glycol, polyglyceryl-3 methylglucose distearate, bisabolol, allantoin, niacinamide, silybin, citrus reticulata (tangerine) fruit extract, glycine soja (soybean) extract, phytosteryl/octyldodecyl lauroyl glutamate, hydroxypinacolone retinoate, retinol, tetradecyl aminobutyroyl valylaminobutyric urea trifluoroacetate, cyclopeptide-161, palmitoyl tripeptide-5, linum usitatissimum(linseed) seed extract, dipalmitoyl hydroxyproline, ascorbyl palmitate, madecassoside, centella asistica extract, asiaticoside, ceramide NP, ceramide AP, oat beta glucan, phytosphingosine, 4-t-butylcyclohexanol, superoxide dismutase, mauritia flexuosa fruit oil, tocopherol, lecithin, hydroxypropyl methylcellulose stearoxy ether, polysorbate 80, divinyldimethicone/dimethicone crosspolymer, pentaerythrityl tetra-di-t-butyl hydroxyhydrocinnamate, trehalose, sucrose laurate, cholesterol, PEG/PPG/polybutylene glycol-8/5/3 glycerin, hydroxyphenyl propamidobenzoic acid, PEG-40 hydrogenated castor oil, caramel, sorbitan trioleate, ethylhexylglycerin, xanthan gum, disodium EDTA, phenethyl alcohol, parfum.

**Data S5：Human Efficacy Study Design**

Inclusion and exclusion criteria are listed in Supplementary Table S3.

**Supplementary Table S3.** Inclusion and exclusion criteria for the human efficacy study.

| Inclusion criteria | 1. Aged 30-60 with mild photoaged and oily or mixed oily facial skin. 2. Clinical score ≥ 2 for visible wrinkles on the forehead, eyebrow, canthus, under the eyes and cheeks, and clinical score ≥ 1 for visible wrinkles on the nasolabial fold and neck (according to the standard Atlas). 3. Clinical score ≥ 1 for facial pores on the cheeks (according to the group standard). 4. Stratum corneum hydration < 50 C.U., transepidermal water loss > 15 g/h/m^2^ and frontal oil secretion content > 100 μg/cm^2^. |
| --- | --- |
| Exclusion criteria | 1. Subjects who used retinoids (e.g., retinol, retinoic acid, tazarotene, adapalene) within 4 months before enrollment. 2. Pregnant, breastfeeding, or planning pregnancy. 3. History of cosmetic or severe allergies. 4. Subjects with systemic or severe skin diseases. |

**Data S6：Supplementary Statistics of Differentially Expressed Genes**


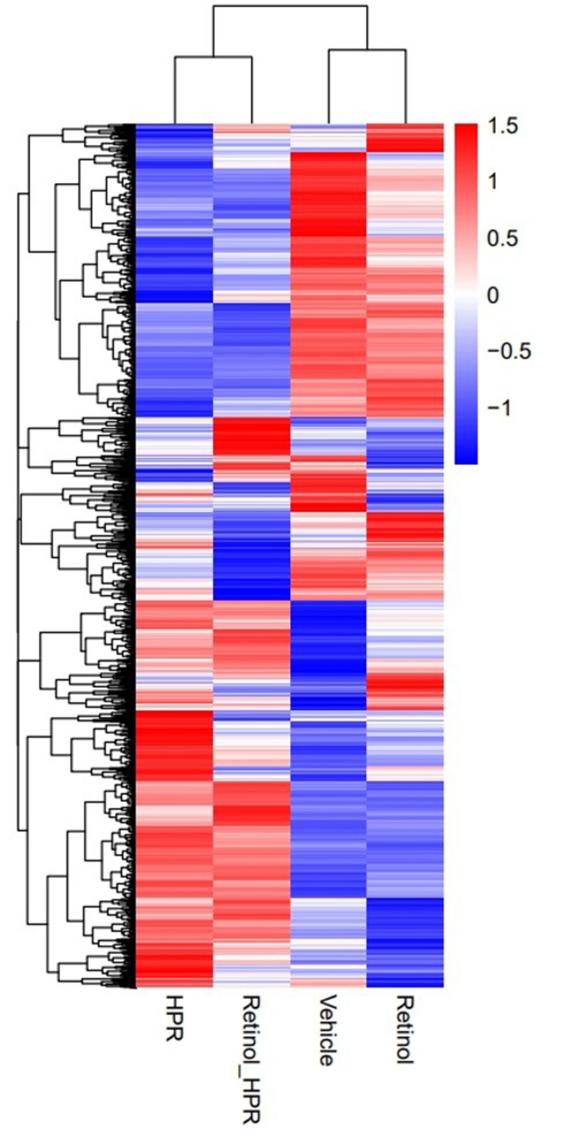


FIGURE S1. Heat map of DEGs. The blue dashed line indicates the threshold line for the differentially expressed gene screening criteria.


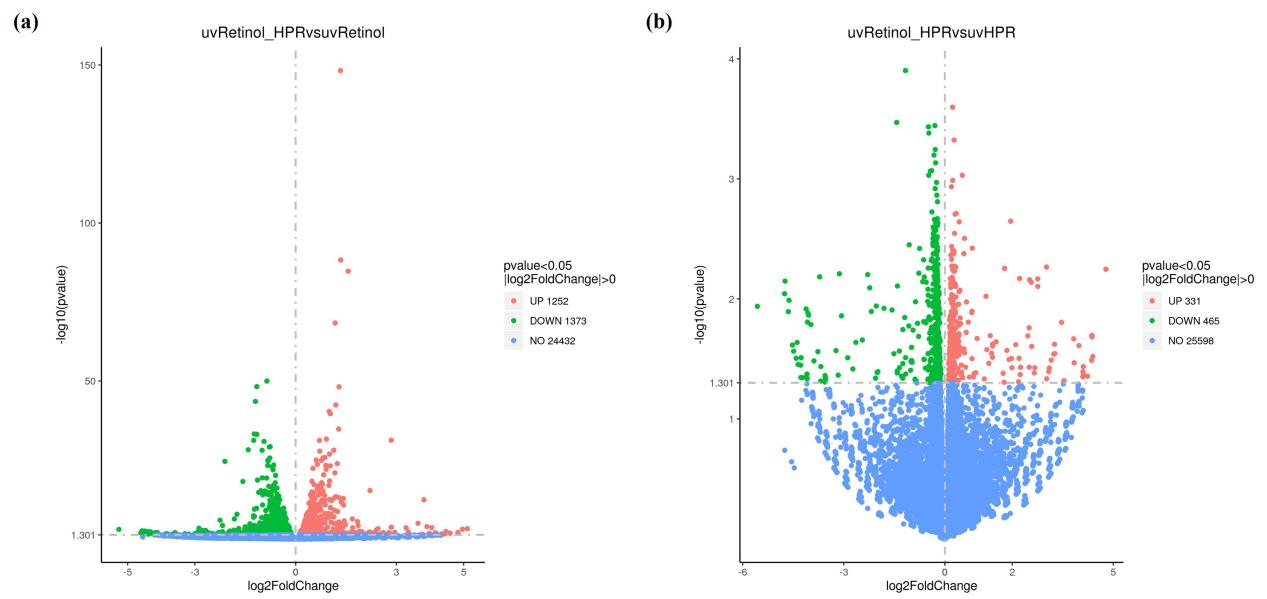


FIGURE S2. Volcano plots of DEGs in Retinol-HPR group vs Retinol group (a) and Retinol-HPR group vs HPR group (b). The blue dashed line indicates the threshold line for the differentially expressed gene screening criteria.

**Data S7：Quantified CYP26B1 Immunoreactivity (IOD/Area) in UV-challenged Human Skin Samples**


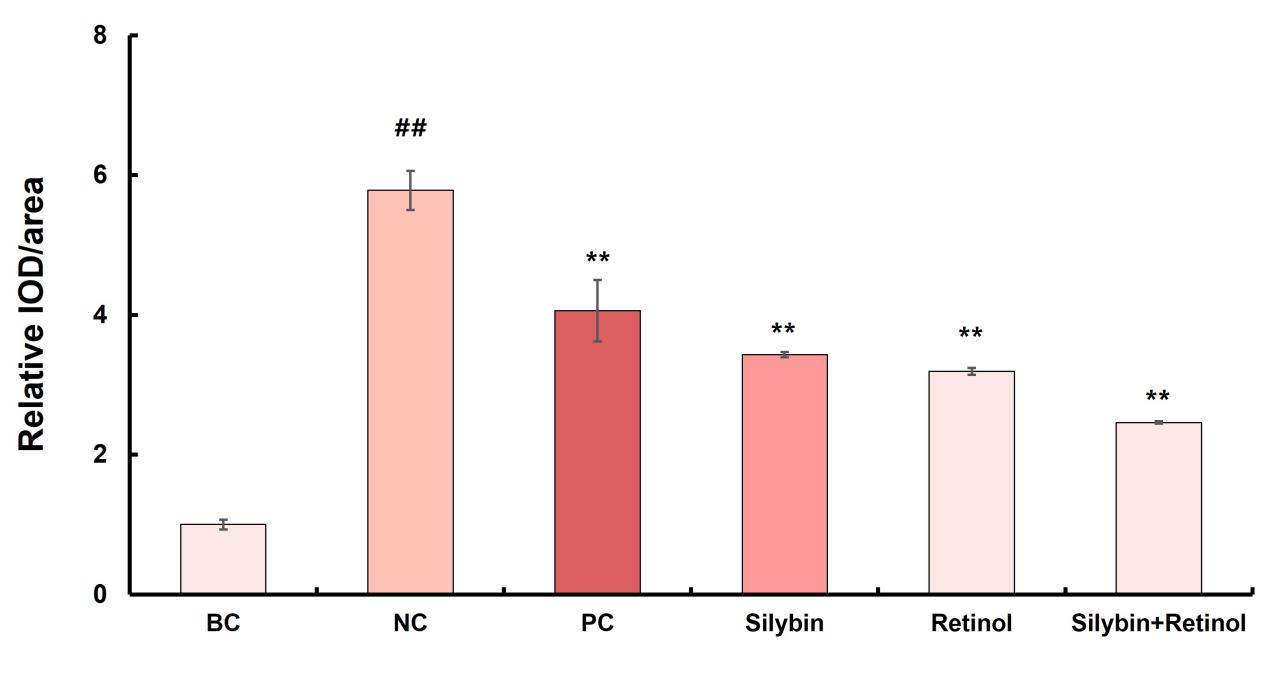


FIGURE S3. CYP26B1 levels were assessed by Immunofluorescence. Data are presented as Relative IOD / Area Mean. IOD stands for integrated optical density, which is a measure of the total fluorescence intensity in a specific area. Data are presented as mean ± SD (## p < 0.01, vs. BC; * p < 0.05, ** p < 0.01, vs.NC).

**Data S8: Approximate concentrations for active components**

Retinol and Hydroxypinacolone Retinoate (HPR): Both retinol and HPR are present at concentrations <0.1%. Literature evidence demonstrates that retinol at 0.1% effectively improves mild photoaged skin in Chinese women [1]. Given the higher sensitivity of Asian skin to retinoids, the sub-0.1% concentration of retinol and HPR avoids the irritation associated with higher doses while maintaining biological activity. The rational ratio of the two retinoids further amplifies their synergistic effects (e.g., TGF-β/Smad pathway activation) without increasing side effects.

Peptides (tetradecyl aminobutyroyl valylaminobutyric urea trifluoroacetate and palmitoyl tripeptide-5): Both peptides are present at concentrations <100 ppm (0.01%). This range is based on the recommended addition level provided by the ingredient suppliers and has been proven to stimulate collagen synthesis. This concentration range can supplement the efficacy of retinol without affecting the stability of the formula.

Silybin: The concentration of silybin is <0.15%. This range is supported by literature [2], which shows that silybin has effective antioxidant and anti-inflammatory effects in ultraviolet-exposed skin models, while enhancing retinoid acid-mediated ECM synthesis. This experiment further verified that this concentration does not interfere with the activity of retinol /HPR or polypeptides, but amplifies the activation of the TGF-β/Smad pathway in the in vitro model.

**References**

[1] Ye Y, Li Y, Xu C. Improvement of mild photoaged facial skin in middle-aged Chinese females by a supramolecular retinol plus acetyl hexapeptide-1 containing essence. *Skin Health and Dis.* 2023;3:ski2. 239.

[2] Boira C, Chapuis E, Lapierre L, et al. Silybum marianum extract: a highly effective natural alternative to retinoids to prevent skin aging without side effects. *J Cosmet Dermatol.* 2025;24:e16613.
